# Efficacy of *Metarhizium anisopliae* and (*E*)–2–hexenal combination using autodissemination technology for the management of the adult greenhouse whitefly, *Trialeurodes vaporariorum* Westwood (Hemiptera: Aleyrodidae)

**DOI:** 10.3389/finsc.2022.991336

**Published:** 2022-09-12

**Authors:** Vongai M. Paradza, Fathiya M. Khamis, Abdullahi A. Yusuf, Sevgan Subramanian, Komivi S. Akutse

**Affiliations:** ^1^ Plant Health Theme, International Centre of Insect Physiology and Ecology (icipe), Nairobi, Kenya; ^2^ Department of Zoology and Entomology, University of Pretoria, Hatfield, South Africa; ^3^ Forestry and Agricultural Biotechnology Institute (FABI), University of Pretoria, Hatfield, South Africa

**Keywords:** autodissemination device, entomopathogenic fungi, fungus–volatile compatibility, lure and infect, biopesticide

## Abstract

The efficiency of an autodissemination technique in controlling adult whiteflies, *Trialeurodes vaporariorum* Westwood (Hemiptera: Aleyrodidae) on tomato, *Solunum lycopersicum* was investigated with previously identified potent fungal isolates of *Metarhizium anisopliae* ICIPE 18, ICIPE 62 and ICIPE 69 under screenhouse or semi-field conditions. The autodissemination device was inoculated with dry conidia of the *M. anisopliae* isolates, while control insects were exposed to a fungus–free device. Sampling for conidia uptake, conidial viability and persistence, and insect mortality was done at 1, 2, 3, 5 and 8 days post–exposure, and collected insects were monitored for mortality over ten days. Overall, mortality was higher in insects exposed to ICIPE 18 (62.8%) and ICIPE 69 (61.8%) than in those exposed to ICIPE 62 (42.6%), with median lethal times, (LT_50_) ranging between 6.73–8.54 days. The control group recorded the lowest mortality rates (18.9%). A general linear reduction in conidial viability with exposure time was observed, although this was more pronounced with *M. anisopliae* ICIPE 62. Insects exposed to *M. anisopliae* ICIPE 69 also recorded the highest conidia uptake, hence selected for further evaluation with a *T. vaporariorum* attractant volatile organic compound, (*E*)–2–hexenal. The volatile inhibited fungal germination in laboratory compatibility tests, therefore, spatial separation of *M. anisopliae* ICIPE 69 and (*E*)–2–hexenal in the autodissemination device was conducted. The inhibitory effects of the volatile were significantly reduced by spatial separation at a distance of 5 cm between the fungus and the volatile, which was found to be more suitable and chosen for the subsequent experiments. Results showed that (*E*)–2–hexenal did not influence conidia uptake by the insects, while fungal viability and the subsequent mortality variations were more related to duration of exposure. The fungus–volatile compatibility demonstrated with spatial separation provides a basis for the optimisation of the volatile formulation to achieve better *T. vaporariorum* suppression with an excellent autodissemination efficiency when used in the management of whiteflies under screenhouse conditions.

## Introduction

Whiteflies (Hemiptera: Aleyrodidae) have emerged as important vectors of several plant viruses that limit crop productivity and cause substantial losses in horticultural production (1). Out of more than 1,550 species that have been described to date, the sweet–potato whitefly, *Bemisia tabaci* Gennadius, and the greenhouse whitefly, *Trialeurodes vaporariorum* Westwood, are recognised as the main vectors of viruses transmitted by whiteflies (2, 3). *Trialeurodes vaporariorum* is a primary pest of several horticultural crops, and a key pest of tomato, cucumber, eggplant, pumpkin and French bean (4, 5). As a polyphagous pest, when farmers practice continuous cropping with successive hosts, which is common in horticultural production systems, the *T. vaporariorum* population builds up rapidly, resulting in high infestations and possible outbreaks (6, 7). In addition, the polyphagous behaviour and persistent high densities predispose whiteflies to excessive applications of different classes of pesticides, resulting in pesticide resistance that makes it difficult to achieve satisfactory control with pesticides (6, 8–10). Adverse effects on both human and ecological health as a result of intensive use of synthetic pesticides against some major arthropod pest species such as whiteflies has been documented (11). These include high pesticide residues on food crops, loss of biodiversity through the elimination of natural enemies and pollinators, and pesticide resistance which subsequently leads to high production costs (12–14). In sub–Saharan Africa, significantly higher pesticide use is common in local and export market cash crops, mainly fruits and vegetables (14). Due to the growing concerns regarding the risks associated with the intensive and indiscriminate use of pesticides in crop protection, calls for the development, adoption and promotion of biological control strategies that strengthen ecological sustainability are becoming increasingly paramount.

The pathogenicity of various entomopathogenic fungi from *Aschersonia*, *Metarhirizium*, *Beauveria*, *Isaria* and *Lecanicillium* genera against whiteflies has been evaluated by several researchers as reviewed by Sani etal. (15). *Metarhizium anisopliae* (Metschnikoff) Sorokin (Hypocreales: Clavicipitaceae) is among these entomopathogenic fungi proven to cause mortality in all stages of *T. vaporariorum* life cycle (16, 17). However, most of the pathogenicity studies have primarily focused on the nymphal stages (9, 11, 18–21), where fungal formulations were mainly applied through leaf spraying and dipping methods (15, 21). There are very limited studies on the adult stage of *T. vaporariorum* where the fungus was used as dry conidia (17). In instances where pathogenicity bioassays were conducted on adults, the method of application was through foliar sprays (22, 23). Similarly, most of the commercial entomopathogenic fungal products available in the market have been formulated for inundative application (24). The majority of these formulations are wettable powders administered as foliar sprays, which remains the most common way to apply entomopathogenic fungi (25, 26). However, because of the high mobility of the pest, spraying sometimes becomes less effective as compared to combining the biopesticide with attractant in a trap through autodissemination approach.

Considering that fungal viability and persistence are crucial for the efficacy of entomopathogenic fungi (21, 27), the application method greatly influences the performance of the pathogen. Spraying poses limitations on efficacy by exposing the fungus on the foliage to abiotic factors like temperature, humidity and ultraviolet radiation, which adversely affect conidial germination, vegetative growth, virulence and pathogenicity of the fungi (28–30). In addition, given both the cryptic and flying behaviour of whiteflies, achieving effective contact by spraying is difficult (31–33). Greater efficiency with spraying may need additional considerations such as specialised sprayers that are unique to each crop’s setting (26). One of the main challenges to the adoption of biopesticides by farmers is their limitation on viability and persistence with field applications (30, 34, 35). Therefore, addressing challenges related to the application system would increase reliability for successful field applications and adoption by growers.

As highlighted by Zanchi etal. (35), adult insects have an advantage of being able to horizontally disseminate the fungus amongst themselves, hence formulating control strategies that are effective for both the adult and immature stages enhances population suppression of the pest (17). Controlling adult insects, however, requires a more targeted application system that enhances efficient delivery of the fungus to its target host. This can be achieved by using an autodissemination device where the fungus is used together with an attractant (“attract and infect/kill”); and this has previously been demonstrated for other insect species [fruit flies – *Ceratitis capitata* and *C. rosa* var. *fasciventris* (36); diamondback moth – *Plutella xylostella* (37); Western flower thrips – *Frankliniella occidentalis* (38); mosquitoes – *Aedes aegypti* (39); pea leafminer – *Liriomyza huidobrensis* (40); polyphagous moth – *Thaumatotibia leucotreta* (41) and the beet worm – *Spoladea recurvalis* (42)].

Research on plant volatile organic compounds in whitefly systems have established their effects on whitefly orientation responses, host seeking behaviour, predator–parasitoids interactions and stimulation of the biosynthesis of defense enzymes that induce plant systemic resistance against pests (43). Semiochemical–based approaches using attractant and repellant plant volatile organic compounds is a developing area of research that seeks to expand the non-chemical approaches that can be adapted for incorporation into various integrated pest management systems against whiteflies (13, 44, 45). From that perspective, the current study explores the application of a semiochemical and entomopathogenic fungi in an integrated biological control approach against *T. vaporariorum*.

Results from our previous study demonstrated the efficacy, spore acquisition and horizontal transmission of *M. anisopliae* ICIPE 18, ICIPE 62 and ICIPE 69 by adults of *T. vaporariorum* (17). The objectives of this current study were therefore to assess the compatibility between the fungal isolates and the attractant, as well as evaluate the efficiency of autodissemination device using dry conidia of the three *M. anisopliae* isolates, ICIPE 18, ICIPE 62 and ICIPE 69 combined with the plant volatile attractant, (*E*)–2–hexenal, for sustainable management of *T. vaporariorum*.

## Materials and methods

### Insects

Experiments were conducted in the laboratory and screenhouses at the International Centre of Insect Physiology and Ecology (*icipe*), Duduville Campus, Nairobi - Kenya (1°13ʹ14.50.ʺ S, 36°53ʹ43.823ʺ E). The whiteflies, *T. vaporariorum* were reared on potted tomato plants, *Solanum lycopersicum* L., cv. Moneymaker and kept in large Perspex cages measuring 40 cm × 60 cm × 80 cm, which were ventilated on the sides with fine netting material. Upon adult emergence, new plants were introduced weekly for oviposition, removed thereafter, and placed into a new cage where the nymphs were left to develop into adults (~ 4 weeks). After wards, a new cage was started with the newly emerged insects from the oldest cage, and new plants were introduced for oviposition. This procedure was repeated in succession for all the other cages, where newly emerged *T. vaporariorum* adults, ≤ 5 days old, were used for the bioassays (46).

### Mass production of the fungus

Three *Metarhizium anisopliae* isolates, ICIPE 18, ICIPE 62 and ICIPE 69 were selected as the most potent against the adults of *T. vaporariorum* based on the findings of our previous study (17). The isolates obtained from the Arthropod Germplasm Centre at *icipe* were mass produced using whole rice as the substrate. Two kilograms of rice, in Milner bags (60 cm long × 35 cm wide) was autoclaved for 1 hour at 121°C, cooled and then inoculated with a 3–day–old culture of the fungal blastospores. The liquid culture for inoculating the rice was obtained by inoculating 50 mL sterilised liquid broth (peptone 15 g, yeast extract 30 g and glucose 30 g/L) with a loopful of spores scrapped off a 2–week–old culture previously sub–cultured on Sabouraud Dextrose Agar (SDA) and incubated in darkness at 25 ± 2°C. The broth was incubated on a rotary shaker at 100 rpm for three days. The volume of the incubated liquid culture was made up to 100 mL with sterile distilled water before inoculating the rice. The inoculated rice was incubated in a culture room for 21 days at 26 ± 1°C, 40–70% RH, and emptied into basins and left to dry for five days. Dry spores were harvested mechanically using a conidia harvester and used immediately. Viability of the fungus was confirmed through a germination test, where 100 µL of a 3 × 10^6^ conidia/mL suspension was spread–plated on three SDA media plates and incubated for 18-20 hours at 25°C. Percentage germination was observed under four microscopic fields covered by cover slips, where 100 conidia were randomly counted under each cover slip using a compound microscope (Leica DM500) at 400× magnification. Conidia with visible germ tubes or which had twice the diameter of the conidium were recorded as viable, and the mean values of the replicates and their standard errors calculated.

### The autodissemination device

**Figure 1:**
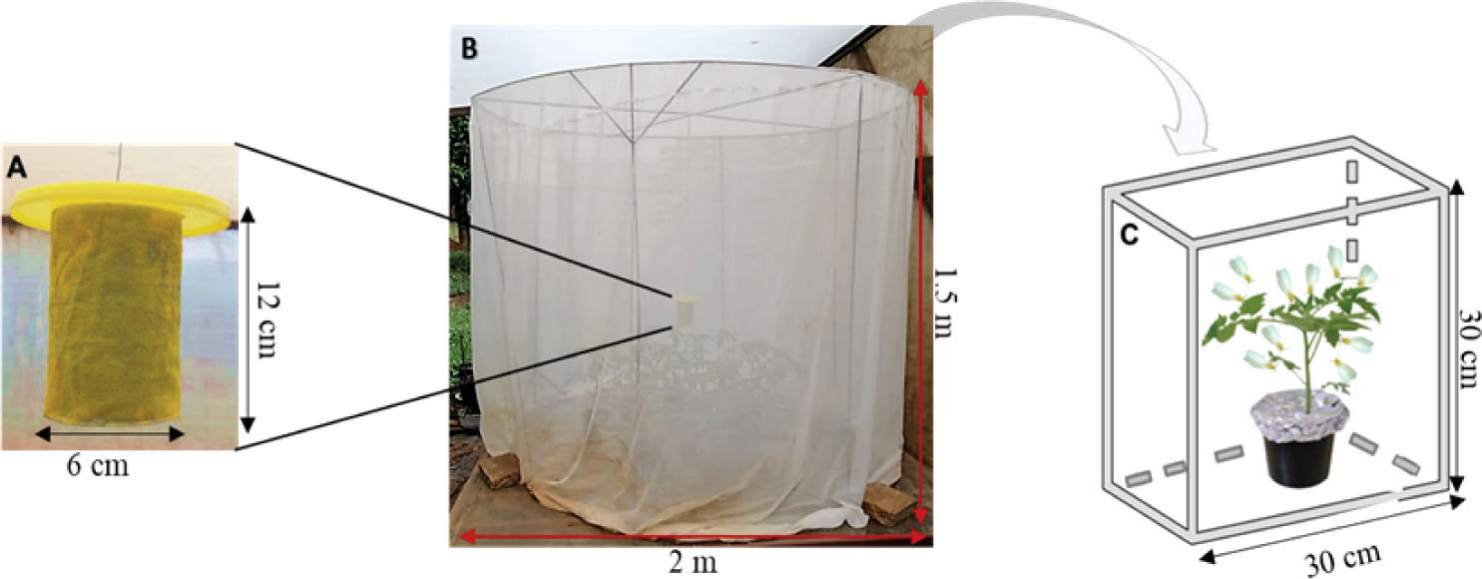
The autodissemination experimental set up: **(A)** the autodissemination device; **(B)** the field cage for insect exposure; **(C)** ventilated plexiglass cage used for mortality observation of insects sampled from the field cage after exposure.

The design of the autodissemination device was similar to the one described by Toledo etal. (47), with slight modifications to its dimensions and features (**Figure 1A**). A cylindrical container (12 cm length and 6 cm diameter) was lined with a yellow velvet material on both the inner and outer surfaces, using a contact adhesive glue. A wire ran through two holes directly opposite each other in the upper section of the device and this was used to secure a lid (12.5 cm diameter) over the cylinder. The bottom of the device was left open. Three grammes (3 g) of dry conidia were applied onto the velvet material on both sides of the device. The velvet material offers good retention capacity of the spores which adhered very well onto this material. A yellow netting, lightly dusted with the dry conidia, was then wrapped around the inoculated velvet material and fastened with pins (40). The lid was also dusted with the fungus on both sides. However, for the control treatment, a fungus-free device was used.

### Evaluation of the autodissemination device

The initial evaluation using only visual cues of the yellow colour of the autodissemination device was done with all the three *M. anisopliae* isolates, ICIPE 18, ICIPE 62 and ICIPE 69. Experiments were conducted under screenhouse conditions in field cages (1.5 m height and 2 m diameter). One caging unit was used per fungal isolate for the evaluation of the autodissemination device. Sixteen five–week–old *Solanum lycopersicum* L. var. Moneymaker plants were placed in a circular arrangement inside the field cage. The plants were heavily infested with approximately ten thousand newly emerged whiteflies taken from the colony. The inoculated autodissemination device was suspended in the centre-top of the cage, 30 cm above the plant canopy (48), while a fungus–free device was used for the control group.

At 1, 2, 3, 5 and 8 days post exposure to the autodissemination device in the field cage (**Figure 1B**), individual whiteflies were randomly sampled from the field cage using an aspirator and released onto a plant in a ventilated Plexiglas cage (30 cm × 30 cm × 30 cm) inside a screenhouse to monitor mortality for ten days (**Figure 1C**), with four replicates per sampling day. The dead whiteflies were surface sterilised in 1% sodium hypochlorite solution, thereafter, rinsed thrice using distilled water. Insects were placed in a Petri dish lined with a moist filter paper for mycosis examination. Percentage of the mycosis was determined by the presence/absence of fungal growth of the inoculated fungus species on the surface of the cadavers. The experiments were repeated twice for each treatment.

#### Assessment of conidial viability and persistence

Conidial viability and persistence were evaluated by carrying out germination tests on conidia collected from the autodissemination devices from each of the treatments at 1, 2, 3, 5 and 8 days after exposure. Four moist cotton buds were used per replicate to collect conidia from different positions of the autodissemination device. The cotton bud with the spores was placed in a universal bottle containing 10 mL of 0.05% Triton X-100 water, vortexed then spread–plated on SDA plates (38) in four replicates. The conidia germination counts were done as described for the determination of viability for the mass production of the fungus in section 2.2.

##### Conidia acquisition by insects

On each sampling day, ten unsexed insects were randomly picked for evaluation of conidia uptake (40). Insects were individually transferred to Eppendorf tubes containing 100µL of 0.05% Triton X-100 water and vortexed to dislodge conidia (36, 42). The concentration of the conidia was determined by counting the number of spores using an Improved Neubauer haemocytometer (VWR International, USA) as described by Inglis etal. (49).

### Compatibility between the fungus and (*E*)–2–hexenal

The plant volatile organic compound, (*E*)–2–hexenal (98% purity, Sigma-Aldrich) used in this study was chosen based on results from a study conducted by Li etal. (50) which identified the volatile as a strong attractant for *B. tabaci* adults at 100× and 1000× dilution. For the analysis of compatibility between *M. anisopliae* isolates ICIPE 18, ICIPE 62 and ICIPE 69 and (*E*)–2–hexenal, viability of the fungus was assessed at two concentrations; 100× and 1000× dilution of (*E*)–2–hexenal using hexane as a solvent at three volumes; 3, 4 and 5 mL in the laboratory. Spores were filtered from a 10 mL (1 × 10^8^ conidia/mL) fungal suspension and retained on a nitrocellulose filter membrane (diameter 47 mm, pore size 0.45 µm, Sigma Chemicals) after filtration using a vacuum filter holder (51). The nitrocellulose membranes were left to dry in a laminar flow cabinet before being transferred to a five–litre glass desiccator. For each volume and concentration, a glass vial was placed in a desiccator with five fungus treated membranes. At day 1, 2, 3, 5 and 8 post exposure, a single membrane was removed, transferred into 10 mL of sterile 0.05% Triton X-100 water and vortexed in order to dislodge conidia. A 100 µL aliquot drawn from the suspension was spread–plated on an SDA plate. Germination was examined 18-20 hours post incubation at 25 ± 2°C. Each membrane was considered a replicate for each corresponding exposure time and each treatment had three replicates/desiccator. The experiment was set up as a completely randomised design.

### Spatial separation of the fungus and the volatile

The above bioassays indicated the same level of compatibility with the volatile for all the isolates at 1000× dilution, while a higher conidia uptake and mortality were recorded with *M. anisopliae* ICIPE 69 than the other isolates, thus it was selected for the subsequent assays with (*E*)–2–hexenal. Therefore, an experiment was set up to reduce the inhibitory effect on conidial germination by spatially separating the volatile and the fungus in the autodissemination device. A glass vial with (*E*)–2–hexenal was placed at three different positions; 0 cm (directly below the device); 5 cm and 10 cm below the autodissemination device. A device with fungus but without (*E*)–2–hexenal was treated as the control (42). The treatments were separated from each other by 10 m and set up in screenhouses and replicated thrice.

#### Evaluation of *Metarhizium anisopliae* ICIPE 69 with (*E*)–2–hexenal in the autodissemination device

Five millilitres of a 1000× dilution of (*E*)–2–hexenal was placed in a 8 mL glass vial (50). The vial was hung from the lid and suspended 5 cm below the autodissemination device inoculated with *M. anisopliae* ICIPE 69. No replacement of the volatile was done once the vial was empty. The viability/persistence of *M. anisopliae* ICIPE 69 was assessed at day 1, 2, 3, 5 and 8 days post–exposure, where moist cotton buds were used to collect spores from the different positions of the replicate autodissemination devices as described above. The cotton buds were then suspended in 10 mL of 0.05% Triton X-100 water and vortexed. A 100 µL aliquot of the suspension was spread–plated on an SDA plate and incubated at 25 ± 2°C for 18-20 hours. Conidial germination was determined as described in section 2.2.

### Data analyses

All the conidial viability/persistence data were analysed using a logistic linear mixed model, (GLMM), with the function *glmer* for the binomial family, where treatments and sampling day were kept as fixed effects while subject (replicate) was treated as the random effect (42, 52). Whitefly mortality data for the different treatments were analysed using a Generalized Linear Model (GLM) assuming a binomial distribution error, and the Probit model in the MASS package was used for the estimation of median lethal time (LT_50_) for each sampling day. The presence/absence binary mycosis data was analysed by GLM with a binomial distribution as the response variable, while treatments and sampling day were the explanatory variables (35). Data on conidial counts based on the different sampling days were fitted into GLM using Poisson regression with treatment and sampling day being the explanatory variables. When means were significantly different among the treatments, a multiple comparison *post hoc* test was performed with the function *emmeans* at 5% significance level (53).

Comparison of treatments with and without the plant volatile were done in two ways; for individual sampling times and for the overall means across the eight days. For individual sampling times, data were first checked for normality using the Shapiro–Wilk test followed by an unpaired Student’s t-test (mortality, conidial viability, and mycosis). Where data showed a non–normal distribution (conidia uptake counts), a two-tailed Mann–Whitney U test was used (54). Data for the overall eight–day means exhibited a non–normal distribution, therefore comparisons for all the variables were analysed with a two-tailed Mann–Whitney U test. All statistical analyses were performed with R version 4.0.5 (55).

## Results

### Fungal viability and persistence in the autodissemination device

**Figure 2:**
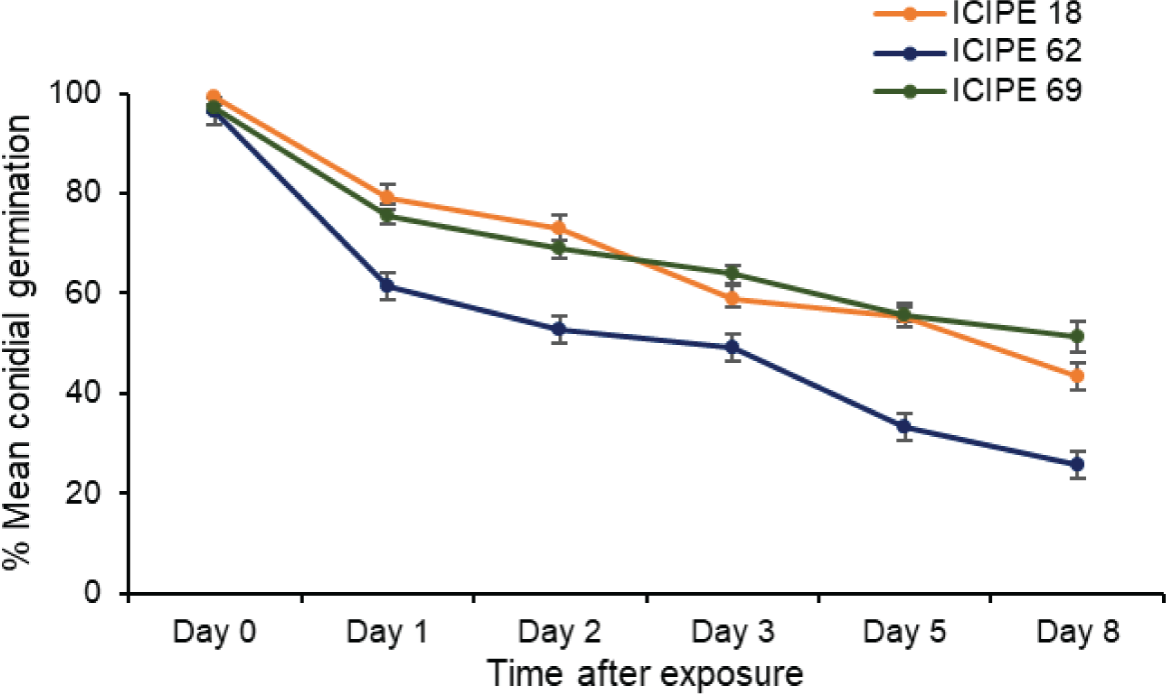
Mean ± standard errors conidial germination of *Metarhizium anisopliae* ICIPE 18, ICIPE 62 and ICIPE 69 in the autodissemination device after placement in the field cage.

There was strong interaction between treatments and duration of exposure (χ^2^ = 141.33, df = 10, P < 0.0001) regarding conidia viability and persistence. There was a general decrease in the viability of the fungus corresponding to an increase in duration of exposure for all the *M. anisopliae* isolates (ICIPE 18, χ^2^ = 1059.4, df = 5, P < 0.0001; ICIPE 62, χ^2^ = 1603.3, df = 5, P < 0.0001; ICIPE 69, χ^2^ = 912.4, df = 5, P < 0.0001). Compared to the other sampling times, the highest decrease in fungus viability occurred 24 hours after exposure, which translated to 20.4, 36.2 and 22.3% loss in viability for *M. anisopliae* ICIPE 18, ICIPE 62 and ICIPE 69 respectively (**Figure 2**). The overall viability of *M. anisopliae* ICIPE 18 and *M. anisopliae* ICIPE 69 was similar and higher (68.2 ± 1.83 and 68.7 ± 1.56%) than *M. anisopliae* ICIPE 62 which significantly exhibited the lowest viability throughout the eight days evaluation period, with an overall germination mean of 53.1 ± 2.23% (**Figure 2**).

### Conidia acquisition after exposure to the autodissemination device

**Figure 3:**
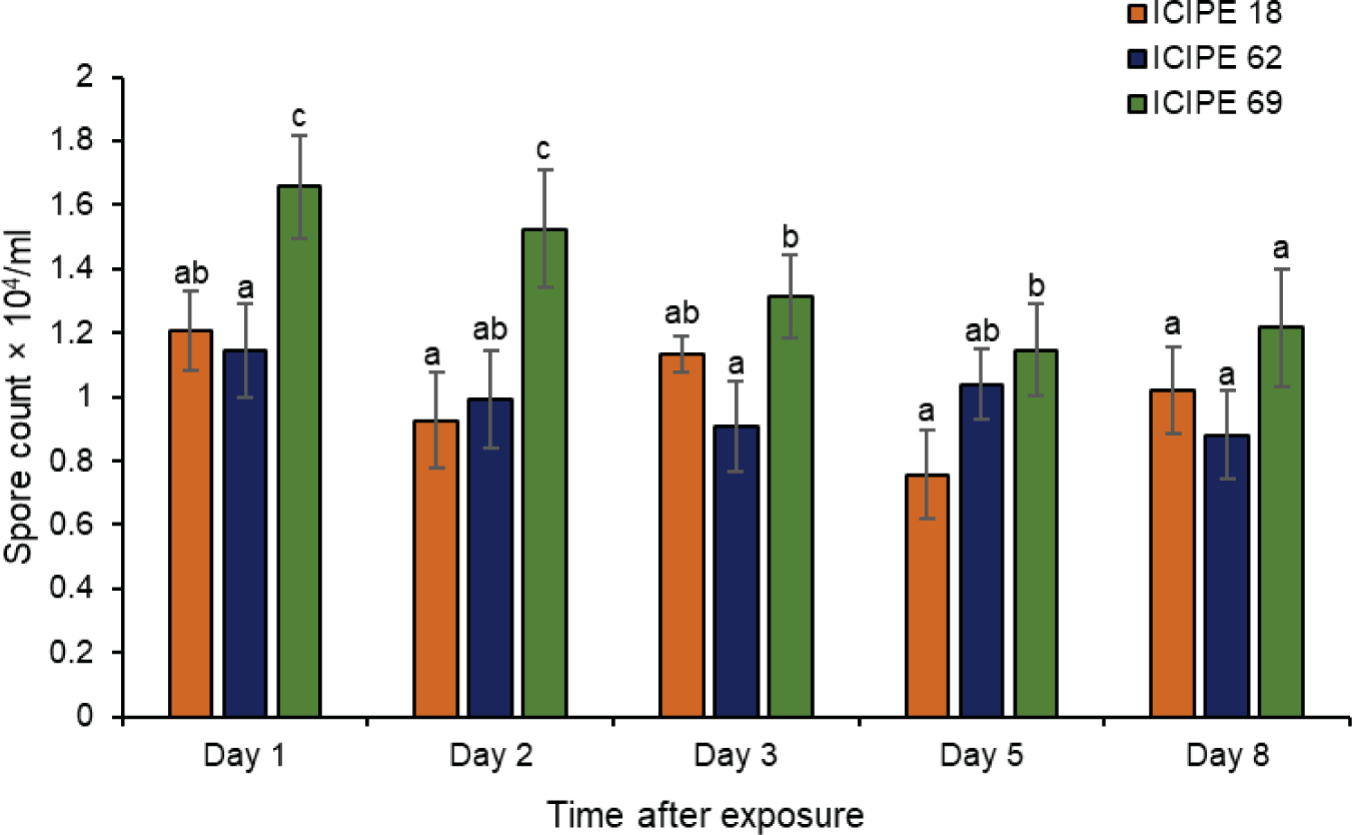
Mean ± standard errors number of conidia picked by individual *Trialeurodes vaporariorum* at different sampling intervals after exposure to autodissemination devices inoculated with *Metarhizium anisopliae* ICIPE 18, ICIPE 62 and ICIPE 69. Significant differences among treatments were denoted by different letters above the bars for each sampling interval (Tukey’s HSD, P < 0.05).

The number of conidia picked by the insects varied significantly among the three isolates (χ^2^ = 7.95, df = 2, P < 0.05). Other than the final day (day 8) which recorded no variations in conidia counts, conidia acquisition by insects exposed to *M. anisopliae* ICIPE 69 was significantly higher than those sampled from *M. anisopliae* ICIPE 18 and *M. anisopliae* ICIPE 62 inoculated autodissemination devices (**Figure 3**). The overall mean conidia number for the five sampling days was highest for *M. anisopliae* ICIPE 69 (1.37 ± 0.16 × 10^4^ conidia/mL) and comparably similar for *M. anisopliae* ICIPE 18 (1 ± 0.12 × 10^4^ conidia/mL) and *M. anisopliae* ICIPE 62 (0.99 ± 0.13 × 10^4^ conidia/mL). The effect of exposure time was not significantly different for each isolate (χ^2^ = 3.80, df = 4, P > 0.05), with the number of fungal spores acquired by individual insects remaining generally constant throughout the sampling days for all the three *M. anisopliae* isolates (ICIPE 18, χ^2^ = 2.54, df = 4, P > 0.05; ICIPE 62, χ^2^ = 0.89, df = 4, P > 0.05; ICIPE 69, χ^2^ = 2.62, df = 4, P > 0.05) (**Figure 3**).

### Insect mortality after exposure to autodissemination devices inoculated with the different *Metarhizium anisopliae* isolates

**Figure 4:**
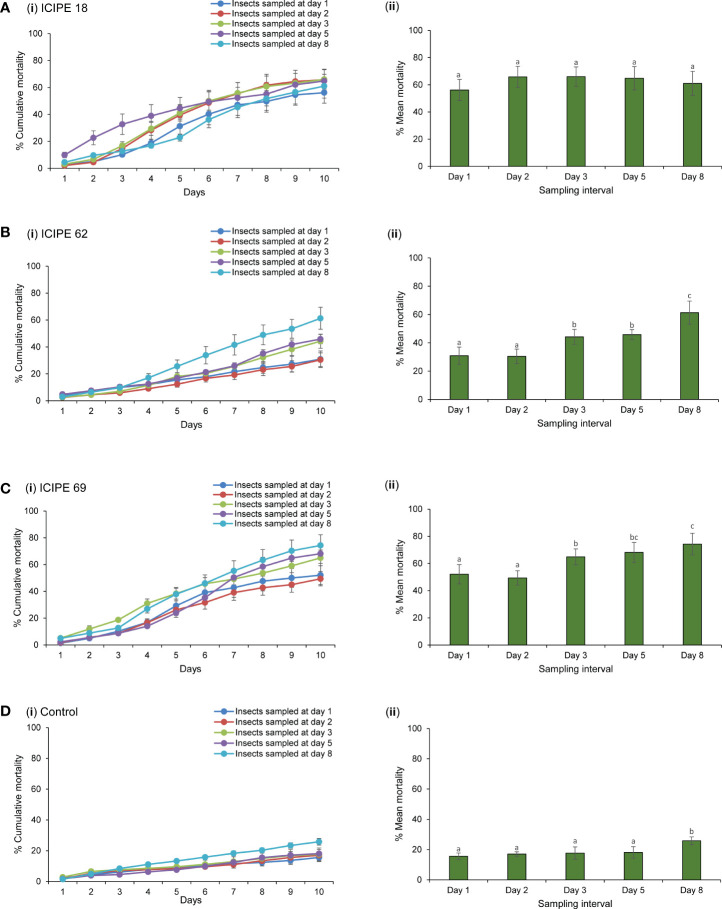
Mortality ± standard errors of adult *Trialeurodes vaporariorum* after exposure to *Metarhizium anisopliae* ICIPE 18, ICIPE 69, ICIPE 62 inoculated autodissemination devices and fungus-free device (control) collected on different sampling intervals. Line graphs **A**(і)–**D**(і) show the cumulative mortality across days for whiteflies sampled on different sampling intervals. Bar graphs **A**(іі)–**D**(іі) show the mean mortality for insects sampled at different time intervals and monitored for 10 days. Means followed by the same letter are not significantly different (Tukey’s HSD test, P < 0.05).

The exposure of insects to inoculated autodissemination devices had a significant effect on their survival or mortality rates. There was no significant interaction between the treatments and duration of exposure denoted as per sampling interval (χ^2^ = 492.4, df = 12, P > 0.05). However, the effects of the main independent variables were highly significant among treatments (χ^2^ = 3312.6, df = 3, P < 0.0001) and the duration of exposure to the fungus (χ^2^ = 376.4, df = 4, P < 0.01). Although an average mortality of 62.8% was recorded from *M. anisopliae* ICIPE 18-treated device, no significant differences (χ^2^ = 46.68, df = 4, P > 0.05) were observed among the various sampling intervals (**Figure 4A**). For the other treatments, mortality was variable across days for *M. anisopliae* ICIPE 62 (χ^2^ = 183.58, df = 4, P < 0.05), *M. anisopliae* ICIPE 69 (χ^2^ = 234.23, df = 4, P < 0.0001) and control (χ^2^ = 56.75, df = 4, P < 0.0001). The mortality of insects exposed to *M. anisopliae* ICIPE 62-treated device remained generally lower than the other two isolates for the first four sampling days with < 50%. However, for insects sampled on the eighth day post exposure, a significant increase led to a mortality of 61.3% (**Figure 4B**). The mortality of insects sampled from the *M. anisopliae* ICIPE 69-treated device showed that at day 1 and 2 post exposure, mortality was significantly lower, (49.4 and 52.1% respectively) than insects sampled at days 3, 5, and 8 (64.9–74.3%) (**Figure 4C**). As expected, insects in the controltreatment recorded the lowest mortality for all the sampling days (**Figure 4D**). In general, *M. anisopliae* ICIPE 18 and *M. anisopliae* ICIPE 69 had comparable averages for the overall mortality rates from the five sapling days (62.8 and 61.8% respectively), followed by *M. anisopliae* ICIPE 62 which averaged 42.6% while the control treatment had the lowest mortality rate of 18.9%.

The median lethal times (LT_50_) were estimated for the three isolates (when mortality > 50%), where the shortest LT_50_ values were obtained at day 5 for *M. anisopliae* ICIPE 18 (6.73 ± 0.45 days) and day 8 for *M. anisopliae* ICIPE 69 (6.77 ± 0.21 days) (**Table 1**). For mycosis, sampling interval did not show any significant influence on the mycosis rates for all the isolates (χ^2^ = 3.07, df = 4, P > 0.05), but only treatment effects explained the differences in the level of mycosis of the cadavers (χ^2^ = 38.78, df = 2, P < 0.0001). There was lower incidence of mycelial growth and sporulation from the cadavers exposed to *M. anisopliae* ICIPE 62–treated device, in comparison to those collected from *M. anisopliae* ICIPE 18 and *M. anisopliae* ICIPE 69 cages which showed no statistical differences among the sampling intervals (**Table 1**). No fungal growth or sporulation was observed on the insects that were incubated from the control group.

**Table 1:** Median lethal time (LT_50_) and mycosis rates of adult *Trialeurodes vaporariorum* collected on different sampling intervals after exposure to autodissemination devices inoculated with *Metarhizium anisopliae* ICIPE 18, ICIPE 62 and ICIPE 69 and monitored over ten days.

Sampling day	ICIPE 18		ICIPE 62		ICIPE 69		
	LT_50_ (days) (95% FL)	% Mycosis(± SE)	LT_50_ (days)(95% FL)	% Mycosis(± SE)	LT_50_ (days) (95% FL)	% Mycosis(± SE)	
1	8.14 ± 0.32	64.8 ± 3.85aB	–	54.5 ± 1.91aA	8.54 ± 0.33		64.2 ± 4.42aB
2	7.03 ± 0.28	65.5 ± 3.54aA	–	57.5 ± 5.04aAB	–		64.1 ± 2.12aA
3	6.97 ± 0.26	64.5 ± 3.19aAB	–	58.2 ± 2.76aA	7.36 ± 0.22		68.3 ± 3.90aB
5	6.73 ± 0.45	64.4 ± 1.93aB	–	52.3 ± 2.12aA	7.57 ± 0.18		64.5 ± 4.22aB
8	8.10 ± 0.29	61.7 ± 1.43aB	8.28 ± 0.28	53.5 ± 1.14aA	6.77 ± 0.21		64.9 ± 3.04aBC

LT_50_ values not estimated (< 50% mortality). FL represents 95% fiducial limits. Within column, means ( ± SE) followed by the same lower case letters and within rows, means ( ± SE) followed by the same upper case letters are not significantly different at P < 0.05 (Tukey’s HSD).

### Compatibility between *Metarhizium anisoplia*e isolates and (*E*)–2–hexenal in the laboratory

**Figure 5:**
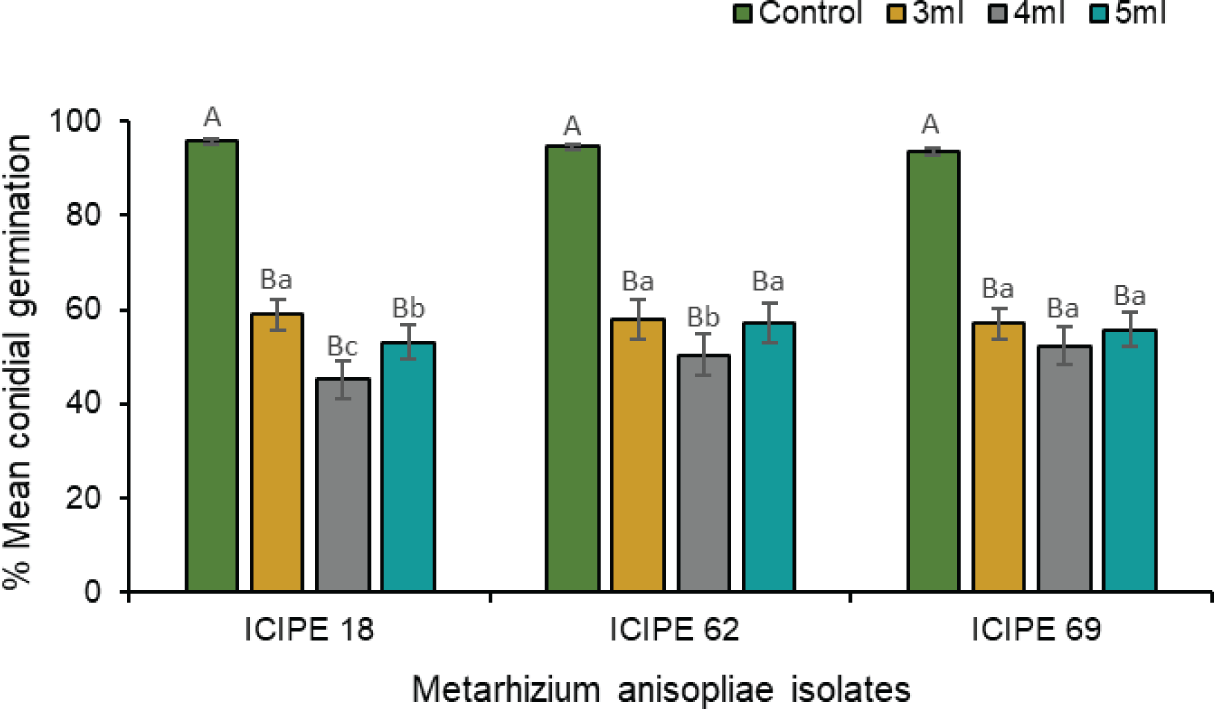
Effects of three volumes of 1000× dilution (*E*)–2–hexenal on the conidial germination of *Metarhizium anisopliae* ICIPE 18, ICIPE 62 and ICIPE 69 eight days post-exposure. Bars denote means ± standard errors and means followed by the same upper–case letters across treatments and lower–case letters within isolate-volume groups are not significantly different (Tukey’s HSD test, P < 0.05).

The laboratory compatibility experiment showed complete inhibition of fungal germination when the isolates were exposed to a 100× dilution of the volatile (*E*)–2–hexanal. At 1000× dilution, the volatile still exerted a pronounced inhibitory effect on conidial germination for all the three isolates compared with their respective control treatments (χ^2^ = 410.84, df = 11, P < 0.0001) (**Figure 5**). However, the amount of tested volatile caused different inhibition levels in the three isolates, with 4 mL exhibiting a stronger inhibitory effect on *M. anisopliae* ICIPE 18 (χ^2^ = 307.11, df = 2, P < 0.0001) and ICIPE 62 (χ^2^ = 88.07, df = 2, P < 0.001) spore germination than 3 mL and 5 mL, while the effect of volatile volume was not significant for *M. anisopliae* ICIPE 69 conidial germination (χ^2^ = 0.20, df = 2, P > 0.05). Viability of spores in the control treatments remained considerably high for all the isolates, with average conidial germination of 93.4–95.7% at 8 days post exposure, while germination counts for volatile exposed *M. anisopliae* treatments ranged between 45.1–59.0% (**Figure 5**).

### Effect of spatial separation of *Metarhizium anisopliae* ICIPE 69 and (*E*)–2–hexenal on conidial viability

The viability of the fungus was significantly affected by the interaction between separation distance and duration of exposure (χ^2^ = 157.46, df = 15, P < 0.0001; **Table 2**). There was general linear reduction in germination in all treatments with increasing duration of exposure (χ^2^ = 2958.1, df = 5, P < 0.0001). In addition, the separation distance had a pronounced effect on germination (χ^2^ = 699.0.38, df = 3, P < 0.0001). The overall viability was lower in all the treatments where the fungus was exposed to volatiles compared to the control, and the level of germination inhibition was dependent on the separation distance. The lowest germination was recorded in the treatment in which the volatile was closest to the fungus (0 cm) (51.0 ± 2.62%), and this effect was reduced with increasing separation distance (**Table 2**). A separation distance of 5 cm was used for evaluation with *M. anisopliae* ICIPE 69 since conidial viability at that distance was not significantly different, (P > 0.05), with that at 10 cm (61.3 ± 2.05 vs. 64.4 ± 1.97%) (**Table 2**).

**Table 2:** Effect of spatial separation of (*E*)–2–hexenal and *Metarhizium anisopliae* ICIPE 69 on conidial germination in the autodissemination device.

Separation distance	Days after exposure								Overall mean
	0	1		2		3	5	8
0 cm	94.7 ± 0.76	55.1 ± 1.81	53.1 ± 1.15		42.2 ± 0.82		33.5 ± 2.37	27.8 ± 2.34	51.0 ± 2.62a
5 cm	94.7 ± 0.76	65.4 ± 1.38	60.8 ± 1.36		58.3 ± 1.21		46.4 ± 1.04	42.6 ± 1.28	61.3 ± 2.05b
10 cm	94.7 ± 0.76	71.3 ± 0.93	64.5 ± 1.32		61.9 ± 1.23		49.9 ± 1.51	44.4 ± 0.82	64.4 ± 1.97bc
Control	94.7 ± 0.76	84.4 ± 1.07	71.0 ± 0.91		66.5 ± 0.77		54.3 ± 0.83	48.9 ± 1.39	69.9 ± 1.93c

Means ± standard errors followed by the same letters with the column are not significantly different at P < 0.05 (Tukey’s HSD).

### Comparative assessment of the efficacy of *Metarhizium anisopliae* ICIPE 69 and (*E*)–2–hexenal combination

**Figure 6:**
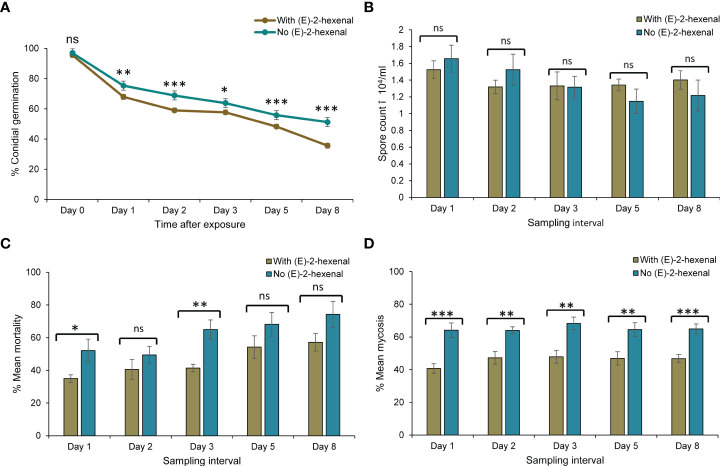
Comparison of response variables between autodissemination devices with and without the volatile ***(E)***–2–hexenal, on *Metarhizium anisopliae* ICIPE 69 and its efficacy. Significant differences between treatments at individual sampling points are indicated on the graphs with asterisks; ^*^P < 0.05, ^**^P < 0.01, ^***^P < 0.001 and ns (non significant, P > 0.05). **(A)** shows conidial viability after the Student’s unpaired t-test with significant differences at Day 1 (t = 3.13, df = 37.6, P < 0.01), Day 2 (t = 4.65, df = 29.1, P < 0.001), Day 3 (t = 2.01, df = 35.0, P < 0.05), Day 5 (t = 4.35, df = 35.5, P < 0.001) and Day 8 (t = 4.64, df = 28.3, P < 0.001); **(B)** shows no significant treatment effect on conidia uptake by the insects on all sampling days following a two tailed Mann–Whitney U tests (P > 0.05); **(C)** shows significant treatment effects on insect mean mortality monitored for ten days for insects collected at Day 1 (t = -2.31, df = 8.42, P < 0.05) and Day 5 (t = -3.83, df = 9.19, P < 0.01) following a Student’s unpaired t-test; and **(D)** indicates significant differences observed on mycosis of the insect cadavers from the two treatments; Day 1 (t = 4.44, df = 12.0, P < 0.001), Day 2 (t = 3.75, df = 10.7, P < 0.01), Day 3 (t = 3.69, df = 14, P < 0.01), Day 5 (t = 2.99, df = 13.9, P < 0.01) and Day 8 (t = 4.65, df = 13.4, P < 0.001) after a Student’s unpaired t-test.

The impact of combining *M. anisopliae* ICIPE 69 and the volatile, (*E*)–2–hexenal on the various response variables, was assessed at a separation distance of 5 cm. The comparative analyses were done per sampling interval as well as on the overall means for the treatments where the autodissemination device did and did not have the volatile (**Figure 6**). The volatile had a significant effect on the viability of the fungus (Mann–Whitney U, W = 9231, P < 0.0001). The presence of the volatile generally lowered the viability of the fungus throughout the evaluation period (**Figure 6A**), leading to a significantly lower conidial germination in the treatment with the volatile (60.8 ± 1.77%) than in the volatile free device (68.6 ± 1.56%). Notably, the presence of the volatile did not affect conidia uptake by the insects over the entire sampling period and the mean spore counts for both treatments did not vary significantly (Mann–Whitney U, W = 4563.5, P > 0.05) (**Figure 6B**). However, despite similar conidia uptake counts by the insects from the two treatments, a significantly lower mortality was observed in insects exposed to an autodissemination device combined with the volatile than a volatile–free treatment at day 1 and day 3 post treatment (**Figure 6C**). The average overall mortality was 26.1% lower in the treatment with the volatile compared to (*E*)–2–hexenal–free treatment (Mann–Whitney U, W = 1175, P < 0.001). The shortest LT_50_ for both treatments were on day 8, although it was shorter for the volatile–free treatment (6.77 ± 0.21 vs. 9.05 ± 0.31 days). Similarly, the mycosis rates in insects exposed to the volatile plus the fungus were significantly lower than in volatile–free treatment, and this trend was consistent for all the days (Mann–Whitney U, W = 1462, P < 0.0001) (**Figure 6D**). Therefore, though considerable compatibility between the attractant and fungus was achieved through spatial separation, the application of (*E*)–2–hexenal still did not improve the efficacy of *M. anisopliae* ICIPE 69 using the autodissemination technology.

## Discussion

The study showed the development and deployment of a simple and effective autodissemination device and its applicability as an efficient entomopathogenic fungi application method against adult whiteflies. The autodissemination device was efficient in attracting insects and allowed for conidia transfer, causing significantly higher mortality rates in the treatments in which the insects were exposed to the fungus, as compared to fungus free control insects. The findings from the study further demonstrated the influence of different factors, mainly related to pathogen virulence, viability and persistence, conidia uptake and mortality on the overall performance of the autodissemination device. However, further results showed that the presence of an attractant plant volatile (*E*)–2–hexenal did not enhance the performance of the autodissemination device.

An autodissemination device allows for the dispersion of conidia by the target insects (32, 56, 57). Insects are attracted towards the device (inoculated with the entomopathogenic fungus), where they pick up the conidia and subsequently spread the propagules to their conspecifics, in a “lure and infect” manner (37, 39). This delivery system presents many advantages; that includes the use of fewer conidia making it more economically viable than innundative application systems, it is simple and easy to set up and has greater persistence of the fungus as it provides some sheltering from the direct exposure to environmental conditions such as temperature/UV and rainfall (42). Additionally, high specificity is attained when the device is used in combination with an attractant lure (57–59).

The autodissemination device in the study was designed with features to enhance both visual and olfactory stimuli since they are major factors in whitefly behaviour. Visual cues in whiteflies are important in long distance responses while plant volatiles facilitate short distance orientation (60, 61). The designed autodissemination device was yellow, based on the colour preference of *T. vaporariorum* adults (62–64). Pinto-Zevallos and Vänninen (48) reported that using cylindrical yellow sticky traps resulted to higher whitefly catches compared to other shapes, hence the same shape was adopted. A yellow velvet material was selected for inoculation because of its high spore retention capacity (40). However, due to the darker pigmentation of the *M*. *anisopliae* conidia (dark green), a yellow netting material was used over the inoculated velvet material to bring out the desired yellow colour.

Combining the entomopathogenic fungus with a plant volatile organic compound was intended to improve the device’s efficiency by adding the odour stimuli. However, a series of optimisations were necessary since combining (*E*)–2–hexenal and *M. anisopliae* ICIPE 69 in an autodissemination device has not been reported elsewhere. Li etal. (50) showed that attraction of *B. tabaci* adults by (*E*)–2–hexenal was concentration dependant. A 100× and 1000× dilution caused 76 and 70% attraction respectively, indicating a stronger response at a higher concentration. For our bioassays, however, a working concentration of 1000× dilution was adopted following laboratory results that showed complete inhibition of conidial germination by (*E*)–2–hexenal at 100× dilution. Although the fungus exhibited a volume–dependant germination rate for *M. anisopliae* ICIPE 18 and ICIPE 62, there were no differences on the overall germination scores over eight days for *M. anisopliae* ICIPE 69, hence the volume that was adopted for bioassays was five millilitres. Inhibition of *M. anisopliae* conidial germination by (*E*)–2–hexenal *via* both direct contact and fumigation has previously been reported (65). To reduce the fungicidal effects of the volatile, spatial separation of the volatile from the fungus was initially assessed at three separation distances of 0, 5 and 10 cm. Based on the results, the evaluation of the compatibility efficacy of the fungus was then conducted using a separation distance of 5 cm since conidial viability at that distance did not differ significantly from that at 10 cm. Since the aim was to have the volatile attract insects towards the inoculated part of the autodissemination device, a shorter distance between the fungus and the volatile also presented an advantage. Although the spatial separation did not eliminate the antifungal effects of the volatile completely, there was significant improvement in fungal viability to levels comparable to the control treatment, similar to findings by Mfuti et al. (66) and Opisa etal. (42).

In consideration of results reported by Li etal. (50) that showed the efficacy of (*E*)–2–hexenal as a strong attractant volatile of *B. tabaci*, there was an expectation that combining the autodissemination device with the volatile would be more effective in attracting whiteflies, effect higher conidia uptake and lead to higher mortality rates. Contrary to this expectation, however, conidia uptake by insects in the presence or absence of the volatile was similar. It is therefore, possible that visits to the autodissemination device by the insects were predominantly influenced by visual cues, the yellow colour of the device. It has been shown that visual cues override volatile/odour cues in whitefly behavioural responses (6, 45, 67). There is also a possibility that the variation in the response to the volatile could be due to species variation, *B. tabaci* used by Li etal. (50) versus *T. vaporariorum* used in the current study. However, both species are recognised as extreme generalists, with a preference for diverse volatile blends and are capable of detecting different volatiles specific to many plants (44, 68). For this reason, we could speculate that species variation may have played a minor role.

There was marked differences in conidial viability of the three different *M. anisopliae* isolates. Exposure of entomopathogenic fungi to the natural environment negatively affects viability due to the differential effects of various abiotic factors. Temperature affects the germination and growth of the fungus, humidity is important for the germination rate of the fungus on the insect’s body and subsequent infection, while direct sunlight exposes the fungus to ultra-violet (UV) radicals that cause alteration in its DNA structure (29, 69). Different fungal species and isolates of the same species have been shown to have variable UV tolerance and thermotolerance levels (70), and this has also been reported for different *M. anisopliae* isolates (71–73). *Metarhizium anisopliae* ICIPE 18 and *M. anisopliae* ICIPE 69 exhibited better and similar environmental fitness with respect to fungal viability and persistence than *M. anisopliae* ICIPE 62. The resulting mortality observed with the two isolates, likewise, were higher than with *M. anisopliae* ICIPE 62.

Similarly, in a follow up experiment with *M. anisopliae* ICIPE 69 and (*E*)–2–hexenal, mortality was higher in a volatile–free treatment than when the autodissemination device was used in combination with the volatile at a 5 cm separation distance. Similar results were reported for bed bugs, *Cimex lectularius* L. (Hemiptera: Cimicidae) where lower mortality was recorded in insects treated with *M. anisopliae* previously exposed to (*E*)–2–octenal (a compound structurally related to (*E*)–2–hexenal), than in treatments where conidia without the aldehyde were used (65). The inhibitory effects of (*E*)–2–hexenal on conidial germination and vegetative growth have also been previously demonstrated by da Silva et al. (74) in *M. anisopliae*, *B. bassiana* (75) and some bacterial strains (76). The fungistatic effects of (*E*)–2–hexenal were reported to be a result of the reactions between the aldehyde’s highly reactive side chains with the nucleophile, sulfhydryl, amino and hydroxyl groups of the fungus through addition and condensation reactions (76). These reactions induce cell damage which impairs the functions of the membrane associated proteins and alters cell permeability resulting in reduced viability. In our current study, in both scenarios, lower mortality rates seemed related to lower fungal viability. Based on this observation, we hypothesised that the lower mortality was as a result of lower fungal viability, since only viable propagules can warrant effective infection which leads to mortality.

In reference to conidia uptake, there was a consistent presence of conidia on the insects that were randomly sampled throughout the evaluation period for all the isolates. There are different possible ways through which conidia acquisition by insects could have occurred; spore transfer following visits to the autodissemination device, horizontal transmission between contaminated and clean individuals or conidia retained by the insects after conidia acquisition. Spore retention and horizontal transmission by the adults of *T. vaporariorum* for up to 72 hours was reported in our previous study (17). Considering that the autodissemination device was the first source of inoculum, the evidence of the presence of conidia on the insects is important as it validates the efficiency of the device in delivering the pathogen. As pointed out by Tol etal. (77), an efficient autodissemination device allows for easy conidia acquisition by insects upon contact and the contaminated adults then disperse the infective propagules to their conspecifics (39). The relatively long median lethal times (LT_50_) recorded (6.73–8.54 days) still present an advantage for the role of the insects as dispersal agents; because a longer survival time for an infected insect gives ample time for horizontal transmission (47, 78).

In conclusion, our findings demonstrated the effective control of adult *T. vaporariorum* using autodissemination device with *M. anisopliae* ICIPE 69 under a screenhouse environment. Using both visual and olfactory stimuli present a good approach in devising an efficient control strategy against whiteflies. Although the results demonstrated that application of the attractant, (*E*)–2–hexenal, did not enhance the efficacy of *M. anisopliae* ICIPE 69 and improve the efficiency of the autodissemination device for the management of the adult greenhouse whitefly, the study still provides important baseline information on the volatile’s effects on both the insect and the fungus, and consequently lays a foundation for comparison with future studies. In addition, the results give a basis for the optimisation of this volatile formulation and, therefore, could guide in designing further experiments to improve the overall efficiency of an integrated system where entomopathogenic fungus and an attractant volatile can be combined (through spatial separation) to achieve better *T. vaporariorum* suppression. This information would also compliment ongoing research approaches that seek to exploit the practical application of semiochemicals in crop protection.

## Data availability statement

The original contributions presented in the study are included in the article/Supplementary Material. Further inquiries can be directed to the corresponding author.

## Author contributions

VP, FK, AY, SS and KA: conceptualisation of the research. FK, SS and KA: funding acquisition and resources. VP: investigation, statistical analyses and writing first draft. VP, FK, AY, SS and KA: validation, data interpretation and writing (reviewing and editing). All authors read and approved the submitted manuscript.

## Funding

This research was funded by BioInnovate Africa Phase I project “Promoting smallholder access to fungal biopesticides through Public Private Partnerships in East Africa” (BA/CI/2017-02/PROSAFE), and UK’s Foreign, Commonwealth and Development Office (FCDO) (FCDO Biopesticide Project, B2291A - FCDO - BIOPESTICIDE) through the International Centre of Insect Physiology and Ecology (*icipe*). We thank the German Academic Exchange Service (DAAD) In-Region Postgraduate Scholarship for the financial assistance provided to the first author through African Regional Postgraduate Programme in Insect Science (ARPPIS). The authors gratefully acknowledge the *icipe* core funding provided by UK’s Foreign, Commonwealth and Development Office (FCDO); Swedish International Development Cooperation Agency (Sida); the Swiss Agency for Development and Cooperation (SDC); the Federal Democratic Republic of Ethiopia; and the Government of the Republic of Kenya.

## Acknowledgments

The authors would like to thank Dr. Daisy Salifu for her statistical advice, and Sospeter Wafula, Jane Kimemia and Levi Ombura for their technical assistance.

## Conflict of interest

The authors declare that the research was conducted in the absence of any commercial or financial relationships that could be construed as a potential conflict of interest.

## Publisher’s note

All claims expressed in this article are solely those of the authors and do not necessarily represent those of their affiliated organizations, or those of the publisher, the editors and the reviewers. Any product that may be evaluated in this article, or claim that may be made by its manufacturer, is not guaranteed or endorsed by the publisher.
